# Ki67 as a predictor of poor prognosis in patients with triple-negative breast cancer

**DOI:** 10.3892/ol.2014.2618

**Published:** 2014-10-15

**Authors:** HAITAO LI, XINGHUA HAN, YINGXIN LIU, GUODONG LIU, GUOMIN DONG

**Affiliations:** Department of General Surgery, The Affiliated Qingzhou Hospital of Weifang Medical College, Qingzhou, Shandong 262500, P.R. China

**Keywords:** breast cancer, estrogen receptor, progesterone receptor, human epidermal growth factor receptor 2, Ki67

## Abstract

The aim of the current study was to investigate the expression of the proliferation antigen, Ki67, in triple-negative breast cancer (TNBC) and its correlation with clinicopathological factors. The expression of Ki67 and other biological indicators in 24 cases of TNBC tissues and 178 cases of non-TNBC tissues were detected using immunohistochemistry. Their correlation with the clinicopathological factors were also analyzed using the χ^2^ test. The positive rate of Ki67 expression in TNBC tissues was 83.3%, exhibiting a statistically significant difference when compared with that in non-TNBC tissues (73.0%) (P<0.05). The expression of Ki67 in breast cancer tissue significantly correlated with the tumor size and lymph node metastases; however, no correlation was observed with the age and the clinical stage. Ki67 may be an indicator of poor prognosis in TNBC patients.

## Introduction

Breast cancer is the most common malignancy occurring in females, accounting for 23% of all malignant tumors (1). With the improvement of biomedical technology, the expression of the nuclear proliferating antigen, Ki67, has been observed to reflect the proliferation rate of malignant tumors. It is associated with the development and metastasis of a variety of malignant tumors, as well as with the prognosis of patients (2). Triple-negative breast cancer (TNBC) refers to a type of breast cancer with negative estrogen receptor (ER), negative progesterone receptor (PR) and negative human epidermal growth factor receptor 2 (Her2) expression, accompanied by characteristic pathological features and molecular expression. TNBC is extremely invasive, exhibits a poor prognosis, is insensitive to endocrine therapy and exhibits a certain organ-oriented metastasis. However, to date, no specific targeted medication has been developed (3–8); therefore, an increasing number of studies focusing on this disease are emerging. In the current study, the expression of Ki67 and other biological indicators in 24 cases of TNBC tissues and 178 cases of non-TNBC tissues were detected using immunohistochemistry. A correlation analysis with the clinicopathological factors (age, tumor size, lymph node metastasis and clinical stage) was also performed, so as to explore the association between the expression of Ki67 and the pathological features, the degree of malignancy and the prognosis of the TNBC patients.

## Patients and methods

### General patient information

A total of 202 breast cancer patients with complete data, diagnosed at the Affiliated Qingzhou Hospital of Weifang Medical College (Qingzhou, China) between October 2011 and May 2013, were enrolled in the current study. All patients were female, the age range was 32–75 years (mean, 45 years). None of the patients had received any neoadjuvant chemotherapy, radiotherapy or other anticancer therapy prior to surgery. Following the surgery, pathological staging was established for the tumor tissues. Patients with additional tumors were excluded from this study. According to the TNM staging criteria (2010 edition), established by the American Joint Committee on Cancer (9), 56 cases were in stage I, 40 cases were in stage IIA, 44 cases were in stage IIB and 62 cases were in stage IIIA. On classification according to the histological type, 170 cases were observed with invasive ductal carcinoma and 32 cases exhibited other types, including invasive lobular carcinoma (n=2), intraductal carcinoma (n=6), intraductal carcinoma complicated with lobular carcinoma (n=2), intraductal carcinoma complicated with micro-invasive ductal carcinoma (n=8), medullary carcinoma (n=2), mucinous adenocarcinoma (n=10) and mixed metaplastic carcinoma (n=2). This study was conducted in accordance with the declaration of Helsinki and with approval from the Ethics Committee of the Affiliated Qingzhou Hospital of Weifang Medical College. Written informed consent was obtained from all participants.

### Immunohistochemistry (IHC)

Rabbit anti-human ER and PR polyclonal antibodies, mouse anti-human Her2 monoclonal antibody and ready-to-use mouse anti-human Ki67 monoclonal antibody were obtained from Fuzhou Maxin Biotechnology Co., Ltd., (Fuzhou, China). Tissues were fixed with 10% neutral buffered formalin for 24 h, followed by conventional dehydration, and were subsequently made into paraffin-embedded specimens. Paraffin-embedded tissues were cut to a thickness of 3 μm and placed on APES-coated slides. The slides were used to detect the expression of Ki67, ER, PR and Her2 using the EnVision IHC kit (Dako, Carpinteria, CA, USA).

### Evaluation of immunohistochemistry

Samples were considered to be positive for Ki67 when the proportion of positively stained cells was >5% (−, ≤5%; +, 6–25%; ++, 26–50%; +++, >50%). On positive expression of Ki67, brown, punctate cellular granules were observed in the nucleus of the tumor cells, or occasionally weakly in the cytoplasm. The mean proportion of positive cells was calculated from any five fields by scanning tumor sections at high power using an Olympus CX31 microscope (Olympus Corporation, Tokyo, Japan; magnification, ×400).

The positive expression of ER and PR was identified as previously described (10), when the proportion of positively stained cells was >1%. However, the positive expression of Her2, which exhibited brown, punctate granules in the cellular membrane, was determined according to the guideline recommendation for Her2 determination, established by the American Society of Clinical Oncology and College of American Pathologists (11), where a positive HER2 result is determined as intense membrane staining of 30% of invasive tumor cells. All breast cancer tissues were divided into the triple-negative group (where the expression of ER, PR and Her2 were all observed to negative) and non-triple-negative group (where any of ER, PR and Her2 were positive).

### Statistical analysis

Data were analyzed using SPSS, version 13.0 (SPSS, Inc., Chicago, IL, USA). The χ^2^ test was conducted to analyze the clinicopathological data. P<0.05 was considered to indicate a statistically significant difference.

## Results

### Expression of Ki67 in breast cancer tissues

**Figure 1:**
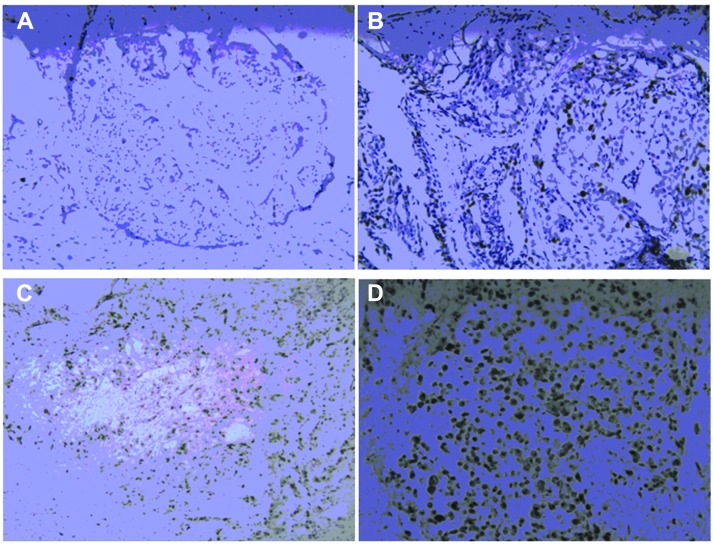
Expression of Ki67 in breast cancer tissues. (A) Negative expression of Ki67 (−); (B) weakly positive expression of Ki67 (+); (C) positive expression of Ki67 (++); and (D) strong positive expression of Ki67 (+++) (A–D, magnification, ×400).

The expression of Ki67 in breast cancer tissues is shown in Fig. 1. The Ki67-positive cells exhibited brown punctate granules in the nucleus. The percentage of positive cells was calculated according to the mean proportion of positive cells in five high-magnification visual fields, and the positive rate of Ki67 expression was determined.

### Expression of Ki67 in TNBC and non-TNBC tissues

In total, 150 of the 202 cases of breast cancer tissues were Ki67-positive, accounting for 74.3%. In the tissue from the 24 cases of TNBC, 20 cases of Ki67-positive expression were identified (83.3%), including 10 cases of strong Ki67 expression. By contrast, in the tissue from the 178 cases of non-TNBC, 130 cases were Ki67-positive, accounting for 73.0%, indicating a statistically significant difference compared with that in the triple-negative group (P=0.000; Table I).

### Correlation between Ki67 expression and clinicopathological data

The expression of Ki67 in breast cancer tissues was significantly correlated with the tumor size and the lymph node metastasis (P<0.05 for both); however, no correlation was identified with the patient age and clinical stage (P>0.05 for both; Table II).

## Discussion

Breast cancer is a highly heterogeneous cancer, exhibiting high diversity in the clinical manifestation, pathology, prognosis, molecular biology and other aspects. With the advancement of molecular biology techniques, breast cancer can now be classified into four subtypes according to the various molecular types, indicating that each different subtype has a corresponding cause (12,13). The 2011 highlights of the St. Gallen International Expert Consensus on the primary therapy of early breast cancer (14–16) defined the breast cancer subtypes immunohistochemically as: Luminal A (ER^+^ and/or PR^+^, Her2^−^ and low Ki67), luminal B (ER^+^ and/or PR^+^, Her2^+^; ER^+^ and/or PR^+^, Her2^−^, high Ki67), Her2-positive (ER^−^, PR^−^ and Her2^+^) and the triple-negative type (ER^−^, PR^−^ and Her2^−^). The molecular typing of breast cancer provides the basis for treatment selection and the prognostic assessment (17). As TNBC accounts for 10–17% of breast cancer cases, and due to the younger onset age, high invasiveness and poor prognosis, it has been widely investigated by researchers (18,19). In the current study, 24 cases (11.9%) of TNBC were identified from the total 202 cases of breast cancer. Furthermore, the TNBC onset ages were younger (median, 47 years; range, 32–75 years), predominantly in premenopausal women, which was similar to that reported in the previous studies.

Ki67 is a nuclear antigen, which exists in proliferative cells. A number of studies have shown that the immune response of Ki67 is closely associated with the cell cycle. It is expressed in the G1, S, G2 and M phase, but not in the G0 phase. Ki67 is weakly expressed in late G1 and early S, and subsequently accumulates in S phase, with a significant increase observed in the latter half of the cell cycle. In mitotic anaphase, the rapid degradation of Ki67 and loss of epitopes has been reported. Furthermore, Ki67 may predict the pathological remission rate in breast cancer patients following neoadjuvant chemotherapy, as an increased Ki67 level following neoadjuvant chemotherapy indicates a poor prognosis (20,21). Therefore, Ki67 is considered to be one of the most significant indicators in detecting the proliferation of tumor cells (22). The expression of Ki67 reliably and quickly reflects the proliferation of malignant cells; it closely correlates with the prediction of the development, metastasis and local recurrence of a variety of malignant tumors (23). Therefore, its positive expression rate is of significance when evaluating the proliferation status of tumor cells, studying the biological behavior of the tumor and when determining the risks.

In the current study, 202 patients with breast cancer were studied, and the overall positive rate of Ki67 expression was 74.3%, which was consistent with the percentage (78%) reported for an Indian population, which was studied by Bhatavdeka *et al* (24). The positive rate of Ki67 expression in TNBC was 83.3%, including 10 cases of strong Ki67 expression. However, it was 73.0% in non-TNBC tissues, which was of statistical significance when compared with that observed in the TNBC tissues (P<0.05). These results suggested that the increased expression of Ki67 may be an important factor for the poor prognosis of TNBC. In addition, Ki67 expression correlated with tumor size and lymph node metastasis in breast cancer, but was not associated with the age and clinical stage. This indicated that the increased expression of Ki67 may predict the increased proliferation of breast cancer cells, enhanced invasiveness, faster growth of the tumor and the high incidence of lymph node metastasis. Therefore, overexpression of Ki67 expression may be an indicator of poor prognosis in TNBC.

## Figures and Tables

**Table I:** Expression of Ki67 in triple-negative and non-triple-negative breast cancer tissues.

		Expression of Ki67				
			
Groups	Patients, n	−	+	++	+++	P-value
Triple-negative	24	4	2	8	10	<0.001
Non-triple-negative	178	48	94	24	12	

**Table II:** Correlation between the expression of Ki67 in breast cancer tissues and the clinicopathological data.

	Triple-negative, n					Non-triple-negative, n				
		
Variables	−	+	++	+++	P-value	−	+	++	+++	P-value
Age, years										
>35	3	1	5	4	0.627	26	50	10	7	0.715
≤35	1	1	3	6		22	44	14	5	
Tumor diameter, cm										
>2	3	1	6	8	0.047	16	52	14	10	0.007
≤2	1	1	2	2		32	42	10	2	
Lymph node metastasis, n										
Positive	1	1	4	6	0.041	11	46	23	6	<0.001
Negative	3	1	4	4		37	48	1	6	
Metastasized lymph nodes, n										
1–3	1	0	1	2	0.039	9	18	6	4	0.011
≥4	0	1	3	4		2	28	17	2	
TNM stage, n										
I	0	1	1	2	0.955	18	26	4	4	0.510
II	1	2	3	5		20	39	10	4	
III	1	1	4	3		10	29	10	4	
